# Three new species of the genus *Probles* Förster (Hymenoptera, Ichneumonidae, Tersilochinae) from South Korea

**DOI:** 10.3897/zookeys.348.6177

**Published:** 2013-11-08

**Authors:** Andrey I. Khalaim, Ekaterina N. Balueva, Ki-Beom Kim, Jong-Wook Lee

**Affiliations:** 1 Zoological Institute, Russian Academy of Sciences, Universitetskaya Emb. 1, St. Petersburg 199034, Russia; 2 Facultad de Ingeniería y Ciencias, Universidad Autónoma de Tamaulipas, Cd. Victoria 87149, Mexico; 3 Department of Life Sciences, Yeungnam University, Gyeongsan, 214-1, Republic of Korea

**Keywords:** *Euporizon*, *Probles*, Tersilochinae, Palearctic region, South Korea, taxonomy, key

## Abstract

Three closely related species of the genus *Probles* Förster, *P. fulgida* **sp. n.**, *P. korusa* **sp. n.** and *P. rukora* **sp. n.**, belong to the subgenus *Euporizon* Horstmann and differ from other Palearctic species of the genus by a combination of long and apically weakly sinuate ovipositor and short temple. These three species are assigned to a newly designated *fulgida* species-group, and a portion of the key for identification of this species-group is provided. Based on the shape of the ovipositor apex, the *fulgida* species-group resemble members of the subgenus *Microdiaparsis* Horstmann but are distinct in having a much shorter temple.

## Introduction

*Probles* is a predominantly Holarctic genus (Nearctic species are mostly undescribed) with about 44 species in the Palearctic region (Yu et al. 2012) and some undescribed species in the Afrotropical and Oriental regions and Australia (Gauld 1984; Khalaim 2007b, 2011). Townes (1971) mentioned a worldwide distribution of this genus but it probably does not occur in South America (Khalaim pers. data). The Palearctic fauna of *Probles* is rather irregularly studied: West European species were revised by Horstmann (1971, 1981; Horstmann and Kolarov 1988), and Palearctic species of five small subgenera, *Microdiaparsis* Horstmann, *Probles* s. str., *Rhynchoprobles* Horstmann and *Rugodiaparsis* Horstmann, were reviewed in two papers by Khalaim (2003, 2007a), whereas the most species rich subgenus, *Euporizon* Horstmann, is virtually unstudied outside Europe. Some species of *Probles* have been recorded as parasitoids of the beetle families Ciidae, Curculionidae, Endomycidae, and Melandryidae in Europe (Horstmann 1971, 1981).

Only two species of *Euporizon*, *Probles (Euporizon) sibirica* Khalaim, 2007 from Mongolia and Russian Siberia and *Probles (Euporizon) vulnifica* Khalaim & Sheng, 2009 from the Palearctic part of China, are known to occur in the East Palearctic region, and one Oriental species, *Probles (Euporizon) vietnamica* Khalaim, 2011, was recently described from Vietnam (Khalaim 2007a, 2011; Khalaim and Sheng 2009). Six more undescribed species of *Euporizon* were reported from South Korea and Vietnam by Khalaim (2011).

Six tersilochine genera, including *Probles*, were found to occur in South Korea; and a key to these genera was provided in our previous paper on Korean Tersilochinae (Balueva et al. 2013). One abundant undescribed species of *Euporizon* comprises over half of the entire Korean material of *Probles* (Balueva et al. unpubl.). In this paper, we describe three closely related new species of *Euporizon* belonging to one species-group (designated here) and provide a portion of the key for identification of these species.

## Materials and methods

This work is based on material of the Ichneumonidae collection of the Yeungnam University (Gyeongsan, South Korea, further YNU). More than 100 specimens of the genus *Probles* have been studied. From this material, three closely related species of the genus *Probles* are described. Most specimens, including all holotypes, are kept at Yeungnam University, with some specimens deposited at the Zoological Institute of the Russian Academy of Sciences, St. Petersburg, Russia (further ZISP) and the Natural History Museum, London, United Kingdom (further BMNH).

Photographs were taken at ZISP using a DFC290 digital camera attached to a Leica MZ16 stereomicroscope; partially focused photographs were combined using Helicon Focus software.

Morphological terminology predominantly follows Townes (1969) with changes according to Khalaim (2011).

## Systematics

### Genus Probles

Förster, 1869

http://species-id.net/wiki/Probles

#### Type species.

*Probles melanarius* Szépligeti, 1895 (= *Porizon erythrostomus* Gravenhorst, 1829).

The genus belongs to the *Tersilochus* genus-group (Horstmann 1981) by having the first metasomal segment with a furrow between the glymma and the ventral part of the postpetiole, and the propodeum usually has a basal area (rarely with basal keel). *Probles* differs from other genera in this genus-group by the well-developed foveate groove of the mesopleuron, which is more or less upcurved anteriorly, elongate thyridial depression, weakly curved hind tibial spurs, and simple tarsal claws. Additional characters for distinguishing *Probles* from other Korean tersilochine genera are given in the key published in our previous paper (Balueva et al. 2013).

### Subgenus Euporizon

Horstmann, 1971

#### Type species.

*Thersilochus rufipes* Holmgren, 1860.

*Euporizon* is the least specialized and the most species rich subgenus of *Probles*, comprising about 36 species in the Palearctic region. This is the only subgenus of *Probles* found in South Korea.

#### Portion of the key to Korean species of Euporizon

**Table:** 

1	Ovipositor weakly sinuate at apex (Figs 10, 18, 24); sheath about 2.5 times as long as first tergite. Temple short, 0.4–0.5 times as long as eye width and very strongly rounded behind eyes in dorsal view (Figs 4, 13, 20). Vertex with sharp and dense punctures on smooth background, distance between punctures mostly less than one diameter of puncture (Fig. 20). Flagellomeres 2 to 6(7) bearing subapical finger-shaped structures on outer surface (Fig. 6, arrows)	*fulgida* species-group, 2
–	Ovipositor not sinuate at apex; sheath usually shorter. Temple longer and/or less rounded behind eyes in dorsal view. Vertex impunctate or finely punctate on smooth or granulate background (distance between punctures greater than one puncture diameter). Formula of finger-shaped structures of flagellum usually not as above	Other species of *Probles (Euporizon)*
2	Foveate groove relatively weak, situated in centre of mesopleuron and not reaching prepectal carina anteriorly (Figs 5, 6). Flagellum with 22 segments (Fig. 2). Clypeus, in lateral view, flat. Wings with distinct yellowish tinge. Ovipositor rather strongly sinuate at apex (Fig. 10)	*Probles (Euporizon) fulgida* sp. n.
–	Foveate groove extending almost entire length of mesopleuron and usually reaching prepectal carina anteriorly (Figs 15, 21). Flagellum with 18–20 segments (Fig. 12). Clypeus, in lateral view, weakly but distinctly convex. Wings with very slight yellowish tinge. Ovipositor weakly sinuate at apex (Figs 18, 24)	3
3	Ovipositor with apex very thin and strongly upcurved (Fig. 18)	*Probles (Euporizon) korusa* sp. n.
–	Ovipositor slightly sinuate at apex, neither especially thin nor strongly upcurved (Fig. 24)	*Probles (Euporizon) rukora* sp. n.

### *fulgida* species-group

**Remarks.** This species-group is designated here for the first time as comprising three Korean species based on characters given in the key. This species-group resembles the subgenus *Microdiaparsis* as both have an apically sinuate ovipositor but is distinct in having a much shorter temple, which is about as long as the eye width in *Microdiaparsis* and only 0.4–0.5 times as long as the eye width in the *fulgida* species-group.

**Description.** Head very strongly constricted and strongly rounded behind eyes in dorsal view (Figs 4, 13, 20); temple short, 0.4–0.5 times as long as eye width. Upper tooth of mandible somewhat longer than lower tooth. Clypeus slightly truncate apically, smooth, punctate in upper part. Malar space 0.7–0.8 times as long as basal width of mandible. Flagellum filiform, usually slightly clavate at apex (Figs 2, 12); subbasal flagellomeres 1.7–1.9 times as long as broad, subapical flagellomeres slightly elongate; flagellomeres 2 to 6(7) bearing apical finger-shaped structures on outer surface (Fig. 6). Vertex with sharp and dense punctures on smooth background, distance between punctures mostly shorter than one diameter of puncture (Fig. 20). Temple smooth and shining, with fine and moderately dense punctures. Hypostomal carina absent. Occipital carina complete.

**Figures 1–5.**
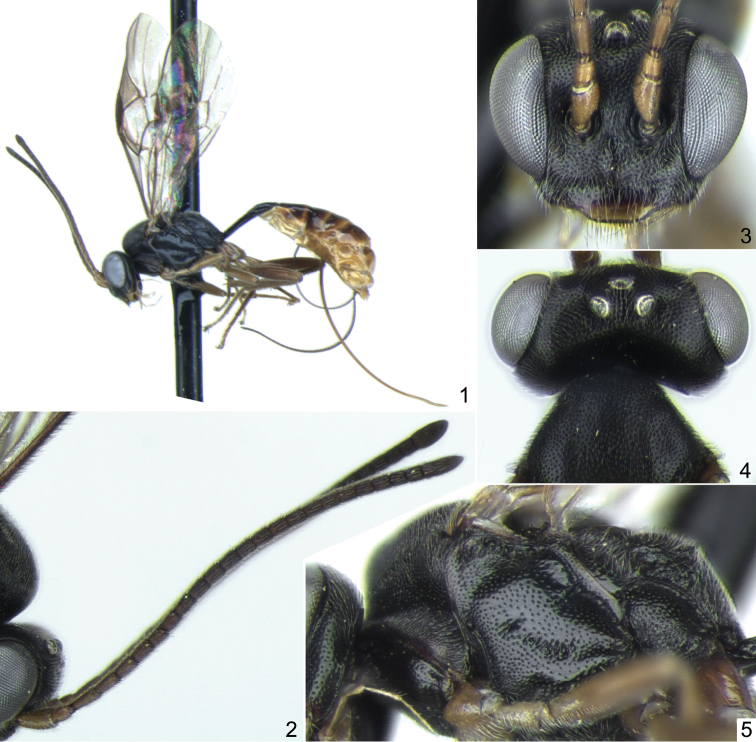
*Probles fulgida* sp. n., ♀, holotype. **1** general habitus, lateral view **2** antennae, lateral view **3** head, frontal view **4** head, dorsal view **5** mesosoma, ventrolateral view.

**Figures 6–10.**
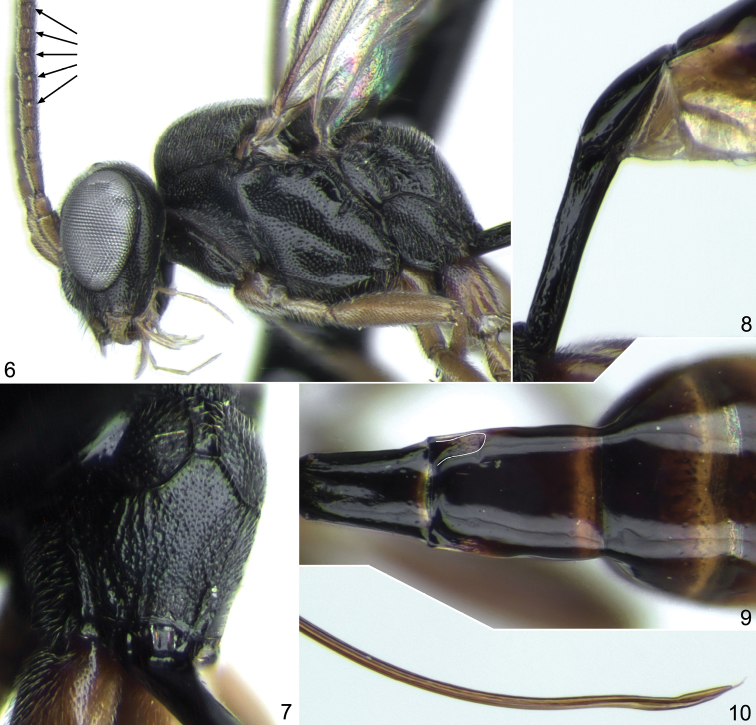
*Probles fulgida* sp. n., ♀, holotype. **6** head and mesosoma, lateral view **7** propodeum, dorso-postero-lateral view **8** first metasomal segment, lateral view **9** second and third segments of metasoma, dorsal view **10** apex of ovipositor, lateral view.

**Figures 11–15.**
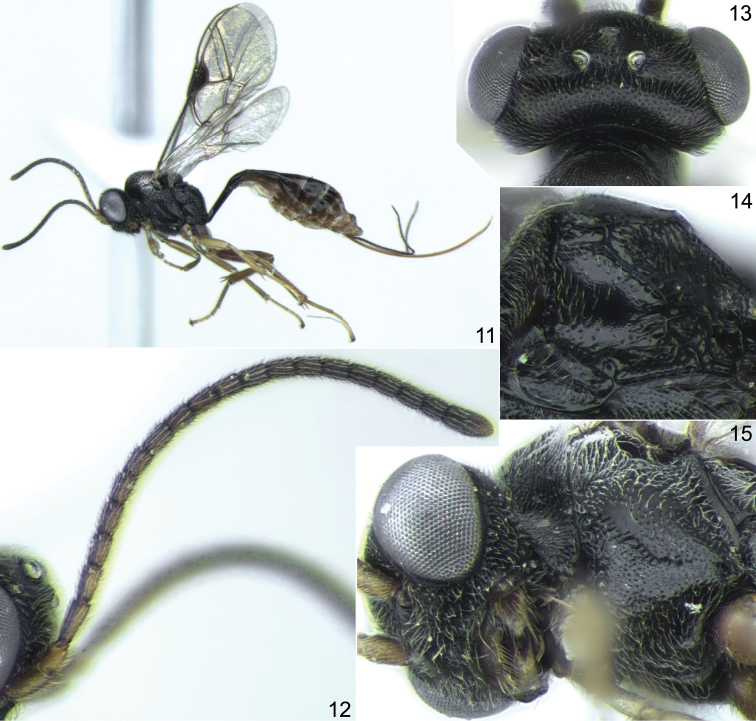
*Probles korusa* sp. n., ♀, holotype (except Fig. **14**). **11** general habitus, lateral view **12** antenna, lateral view **13** head, dorsal view **14** propodeum, dorsolateral view **15** head and mesopleuron, ventrolateral view.

Notaulus with distinct wrinkle adjacent to anterolateral margin of mesoscutum (Fig. 20). Mesoscutum densely punctate, granulate, dull. Scutellum with lateral longitudinal carinae developed in its anterior 0.3–0.4. Foveate groove well developed, S-curved, crenulate (Figs 15, 21). Mesopleuron centrally (above foveate groove) distinctly punctate, smooth and shining between punctures (Figs 5, 15). Propodeum with more or less rectangular, usually slightly widened anteriorly basal area, which is 1.5–2.0 times as long as broad and 0.35–0.5 times as long as apical area (Figs 7, 14, 22). Dorsolateral area finely granulate, sometimes almost smooth centrally, finely punctate or impunctate. Propodeal spiracle separated from pleural carina by 1.0–2.0 times diameter of spiracle. Apical area flat, truncate anteriorly, granulate or uneven, impunctate.

**Figures 16–18.**
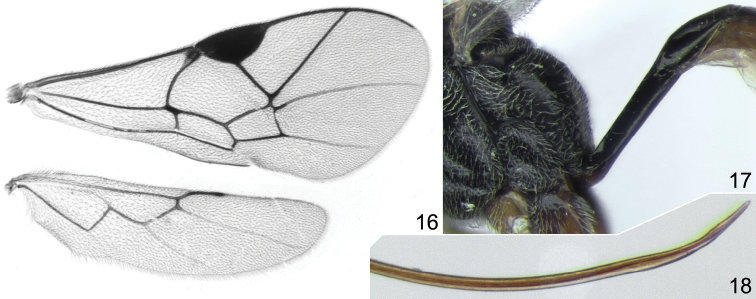
*Probles korusa* sp. n., ♀, paratype (Fig. **16**) and holotype (Figs **17, 18**). **16** wings **17** posterior part of metasoma and first tergite, lateral view **18** apex of ovipositor, lateral view.

Fore wing (Fig. 16) with second recurrent vein distinctly postfurcal; intercubitus about as long as abscissa of cubitus between intercubitus and second recurrent vein. Metacarp not reaching apex of fore wing. First abscissa of radius about 1.5 times as long as width of pterostigma. Postnervulus intercepted below middle. Hind wing (Fig. 16) with nervellus vertical or slightly reclivous.

**Figures 19–24.**
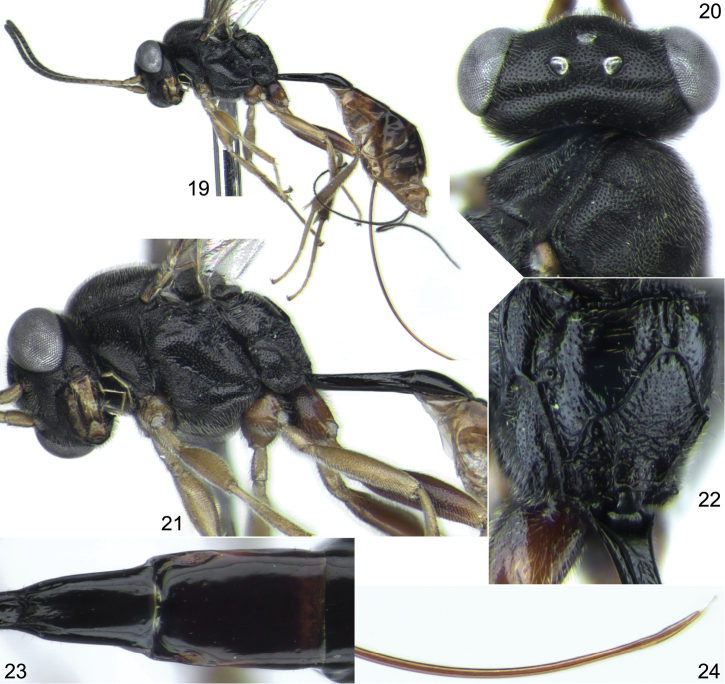
*Probles rukora* sp. n., ♀, holotype. **19** general habitus (without wings), lateral view **20** head and anterior part of mesosoma, dorsal view **21** head, mesosoma and first tergite, lateral view **22** propodeum, dorsolateral view **23** second tergite, dorsal view **24** apex of ovipositor, lateral view.

Legs slender. Hind femur about 4.0 times as long as broad and 0.8–0.85 times as long as tibia. Hind spurs slightly curved at apex. Tarsal claws not pectinate.

First tergite 2.9–4.0 times as long as broad posteriorly; petiole centrally more or less trapeziform in cross-section, distinctly separated from postpetiole in dorsal view. Glymma moderately deep, situated behind centre of first tergite, joining by distinct furrow to ventral part of postpetiole (Figs 8, 17, 21). Second tergite 1.55–1.65 times as long as anteriorly broad (Figs 9, 23). Thyridial depression well developed, deep, about 2.5–3.0 times as long as broad (Figs 9, 23). Ovipositor weakly sinuate at apex (Figs 10, 18, 24); sheath about 2.5 times as long as first tergite.

Head, mesosoma, and first tergite black. Palpi and mandible (teeth reddish black) yellow. Clypeus brownish yellow in lower 0.3–0.4. Scape and pedicel of antenna yellowish; flagellum fuscous, a little paler basally. Tegula yellow to brownish. Pterostigma brown. Legs brownish yellow; hind coxa and femur (sometimes also mid coxa) brown, hind tibia infuscate. Metasoma behind first tergite usually brown, ventrally yellowish, tergites 2 to 5 dorsally usually with more or less distinct yellow band posteriorly (Fig. 9).

**Composition.** This species-group comprises three closely related species, *Probles fulgida* sp. n., *Probles korusa* sp. n. and *Probles rukora* sp. n., occurring in South Korea.

### Probles (Euporizon) fulgida

Khalaim & Balueva sp. n.

http://zoobank.org/0F412ACA-A932-4669-9B03-517C2F45B55B

http://species-id.net/wiki/Probles_fulgida

Figs 1 –10

#### Holotype.

Female (Fig. 1), SOUTH KOREA: Gyeongsangnam-do, Sancheong-gun, Sicheon-myeon, Mt. Jiri, Jangdanggol, 35°20'N, 127°43'E, 11.VIII–8.IX.2001, coll. J.W. Lee (YNU).

#### Comparison.

Differs from the two other members of the *fulgida* species-group, *Probles korusa* sp. n. and *Probles rukora* sp. n., by the weaker and shorter foveate groove of the mesopleuron (Fig. 5), 22-segmented antennal flagellum (Fig. 2), flat clypeus (in lateral view), wings more extensively tinged with yellow, and more strongly sinuate apex of the ovipositor (Fig. 10).

#### Description.

Female: Body length 5.5 mm; fore wing length 3.85 mm. Head with temple almost half as long as eye width in dorsal view (Fig. 4). Clypeus flat in lateral view, smooth, distinctly and densely punctate on upper half (Fig. 3). Malar space 0.7 times as long as basal width of mandible (Fig. 6). Antennal flagellum with 22 segments (Fig. 2); flagellomeres 2 to 7 bear subapical finger-shaped structures on outer surface (Fig. 6). Face and frons very finely granulate, dull, with sharp and dense punctures (Fig. 3). Temple with dense, fine and sharp punctures. Foveate groove relatively weak and short, situated near center of mesopleuron (Fig. 5). Mesopleuron almost entirely sharply and densely punctate, peripherally granulate (Figs 5, 6). Propodeum with basal longitudinal carinae anteriorly indistinct, basal area rectangular, about 1.6 times as long as broad and 0.35 times as long as apical area (Fig. 7). Propodeal spiracle separated from pleural carina by 2.0 times diameter of spiracle. Apical longitudinal carinae mostly indistinct (Fig. 7). Hind femur 4.3 times as long as broad and 0.82 times as long as tibia. First tergite laterally before glymma finely striate, 2.9 times as long as broad posteriorly (Fig. 8). Second tergite 1.55 times as long as anteriorly broad (Fig. 9). Ovipositor distinctly sinuate at apex (Fig. 10); sheath about 2.5 times as long as first tergite. Metasoma behind first tergite extensively brownish yellow ventrally and laterally, tergites 2 to 5 dorsally and dorsolaterally brown (Fig. 1).

Male. Unknown.

#### Distribution.

South Korea.

#### Etymology.

Named from the Latin *fulgidus* (shining, gleaming, glittering).

### Probles (Euporizon) korusa

Khalaim & Kim sp. n.

http://zoobank.org/25EF822B-B580-43F5-AC32-DAA0D6C5336A

http://species-id.net/wiki/Probles_korusa

Figs 11 –18

#### Holotype.

Female (Fig. 11), SOUTH KOREA: Gyeongsangbuk-do, Cheongdo-gun, Unmun mueon, Ssalbawi, 35°38'08"N, 129°01'27"E, 29.VI–10.VII.2012, coll. J.W. Lee (YNU).

#### Paratypes.

SOUTH KOREA: Chungcheongbuk-do: Danyang-gun, Cheondong-ri, Mt. Sobaeksan, 37°00'N, 128°31'E, Malaise trap, 21.VI–6.VII.2006, coll. J.W. Lee, 1 ♀ (YNU). Gyeonggi-do: Yangpyeong, Yongmun, Yeonsu, Mt. Yongmunsan, 324 m, 37°31'48.9"N, 127°34'23.8"E, Malaise trap, 11–25.VI.2009, coll. J.O. Lim, 1 ♀ (YNU). Mt. Yongmunsan, Yeonsu, Yongmun, Yangpyeong, 324 m, 37°31'48.9"N, 127°34'23.8"E, 11–25.VI.2009, coll. J.W. Lim, 1 ♀ (ZISP). Gyeongsangbuk-do: same data as holotype, 3 ♀♀ (YNU, 1 ♀ in ZISP). Jeollabuk-do: Jeongeup-si, Naejang-dong, Geumseong, Malaise trap, 20.VI.2005, coll. D.K. Chung, 1 ♀ (YNU).

#### Additional material.

RUSSIA: Primorskiy reg., 30 km SE of Ussuriysk, mixed forest, 15.VII.2001, coll. S.A. Belokobylskij, 1 ♀ (ZISP).

#### Comparison.

Very similar to *Probles rukora* sp. n. but the ovipositor has the apex thinner and strongly upcurved (Fig. 18), whereas in *Probles rukora* sp. n. the ovipositor is just slightly sinuate apically with the extreme apex neither especially thin nor strongly upcurved (Fig. 24). No other differences were found between these two species but the shape of the ovipositor apex works very well in separating all specimens without intermediate forms and both species are represented in our material by many specimens. Thus, we consider *Probles korusa* sp. n. and *Probles rukora* sp. n. to be distinct species. *Probles korusa* sp. n. also resembles the European species *Probles curvicauda* Horstmann, 1981, which also has a long ovipositor with strongly upcurved apex, but is distinct in having much shorter temple, a wider basal area of the propodeum and a somewhat shorter ovipositor sheath.

#### Description.

Female: Body length 4.5 mm; fore wing length 3.15 mm. Head with temple 0.45 times as long as eye width in dorsal view (Fig. 13). Clypeus weakly convex in lateral view, densely punctate on upper 0.6, finely granulate near upper margin. Malar space about 0.8 times as long as basal width of mandible (Fig. 15). Antennal flagellum with 19–20 segments (19 segments in holotype) (Fig. 12). Face and frons very finely granulate, dull, with sharp and dense punctures. Temple with dense, fine and sharp punctures. Foveate groove long, extending across anterior 0.8 of mesopleuron (Fig. 15). Propodeum with basal area about 1.5 times as long as broad and almost half as long as apical area (Fig. 14); basal longitudinal carinae sometimes indistinct and propodeum with longitudinal wrinkles dorsally. Propodeal spiracle separated from pleural carina by 1.0–2.0 times diameter of spiracle. Apical longitudinal carinae usually weak, anteriorly usually indistinct. Hind femur 4.1 times as long as broad and 0.82 times as long as tibia. First tergite laterally before glymma mostly smooth, 4.0 times as long as broad posteriorly. Second tergite 1.65 times as long as anteriorly broad. Ovipositor weakly sinuate apically, with extreme apex thin and strongly uncurved (Fig. 18); sheath about 2.6 times as long as first tergite. Metasoma behind first tergite predominantly brown, yellowish ventrally, tergites 2–3(4) dorsally dark brown (Fig. 11).

Male. Unknown.

#### Variation.

The holotype has rather weak punctures on the temple. A female from the Russian Far East corresponds well with Korean material of this species but has the vertex conspicuously impressed posteriorly, weak notaulus, and a weaker foveate groove of the mesopleuron, and it may belong to a new species. This specimen is not included in the type series, and study of additional material is required to solve its status.

#### Distribution.

South Korea, Russian Far East (Primorskiy reg.).

#### Etymology.

Combination of initial letters of Korea and Russia, home countries of the participants in this paper.

### Probles (Euporizon) rukora

Khalaim & Lee sp. n.

http://zoobank.org/BE207642-1A2E-4ACB-B9E3-A08CCE61FFE7

http://species-id.net/wiki/Probles_rukora

Figs 19–24

#### Holotype.

Female (Fig. 19), SOUTH KOREA: Chungcheongbuk-do, Boeun-gun, Songnisan, Deopjusameapyoso, 36°32'06"N, 127°49'40"E, 12–21.VI.2007 (YNU).

#### Paratypes.

SOUTH KOREA: Gangwon-do: Inje-gun, irin-muen, Jindong-ri, Jeombongsan, 26.VI–28.VII.2012, coll. J.Y. Park, 1 ♀ (ZISP). Gyeongsangbuk-do: Cheongdo-gun, Unmun-myeon, Mt. Unmunsan, 35°38'45"N, 128°57'33"E, Malaise trap, 1–24.VII.2008, coll. J.W. Lee, 1 ♀ (YNU). Gyeongsangnam-do: Yeongju-si, Punggi-eup, Jungnyeong, 35°53'42.7"N, 128°26'22"E, 12–23.VII.2008, coll. J.M. Kwon, 1 ♀ (YNU).

#### Comparison.

Very similar to *Probles korusa* sp. n. but the ovipositor is very weakly sinuate apically, with the extreme apex neither especially thin nor strongly upcurved (Fig. 24), whereas in *Probles korusa* sp. n. the ovipositor is very thin and strongly upcurved at the extreme apex (Fig. 18). See also Comparison section for *Probles korusa* sp. n.

#### Description.

Female: Body length 5.4 mm. Fore wing length 3.75 mm. Head with temple 0.42 times as long as eye width in dorsal view (Fig. 20). Clypeus weakly convex in lateral view, densely punctate on upper 0.6. Malar space 0.7–0.8 times as long as basal width of mandible. Antennal flagellum with 20 segments. Face and frons very finely granulate, dull, with sharp and dense punctures. Temple with dense and fine punctures. Foveate groove long, extending across anterior 0.8 of mesopleuron (Fig. 21). Propodeum with basal area 1.5–2.0 times as long as broad and 0.37 times as long as apical area (Fig. 22); basal longitudinal carinae sometimes indistinct and propodeum with longitudinal wrinkles dorsally. Propodeal spiracle separated from pleural carina by 1.0–2.0 times diameter of spiracle. Apical longitudinal carinae weak anteriorly. Hind femur 3.9 times as long as broad and 0.84 times as long as tibia. First tergite laterally mostly smooth, before glymma partly striate, 3.8 times as long as broad posteriorly. Second tergite 1.65 times as long as anteriorly broad (Fig. 23). Ovipositor very weakly sinuate apically (Fig. 24); sheath about 2.5 times as long as first tergite. Metasoma behind first tergite predominantly brown, yellowish ventrally, dorsally mostly dark brown (Fig. 19).

Male. Unknown.

#### Distribution.

South Korea.

#### Variation.

This is a rather uniform species with no obvious variation in structure and coloration.

#### Etymology.

Combination of initial letters of Russia and Korea, home countries of the participants in this paper.

## Supplementary Material

XML Treatment for Probles

XML Treatment for Euporizon

XML Treatment for Probles (Euporizon) fulgida

XML Treatment for Probles (Euporizon) korusa

XML Treatment for Probles (Euporizon) rukora
